# Kinetics of IL-7 and IL-15 Levels after Allogeneic Peripheral Blood Stem Cell Transplantation following Nonmyeloablative Conditioning

**DOI:** 10.1371/journal.pone.0055876

**Published:** 2013-02-21

**Authors:** Muriel De Bock, Marianne Fillet, Muriel Hannon, Laurence Seidel, Marie-Paule Merville, André Gothot, Yves Beguin, Frédéric Baron

**Affiliations:** 1 Groupe Interdisciplinaire de Génoprotéomique Appliquée (GIGA)–I^3^, University of Liège, Liège, Belgium; 2 Department of Statistics, University of Liège, Liège, Belgium; 3 Department of Medicine, Division of Hematology, University and Centre Hospitalier Universitaire (CHU) of Liège, Liège, Belgium; CNRS, France

## Abstract

**Background:**

We analysed kinetics of IL-7 and IL-15 levels in 70 patients given peripheral blood stem cells after nonmyeloablative conditioning.

**Methods:**

EDTA-anticoagulated plasma and serum samples were obtained before conditioning and about once per week after transplantation until day 100. Samples were aliquoted and stored at −80°C within 3 hours after collection until measurement of cytokines. IL-7 and IL-15 levels were measured by ELISAs.

**Results:**

Median IL-7 plasma levels remained below 6 pg/L throughout the first 100 days, although IL-7 plasma levels were significantly higher on days 7 (5.1 pg/mL, P = 0.002), 14 (5.2 pg/mL, P<0.001), and 28 (5.1 pg/mL, P = 0.03) (but not thereafter) than before transplantation (median value of 3.8 pg/mL). Median IL-15 serum levels were significantly higher on days 7 (12.5 pg/mL, P<0.001), 14 (10.5 pg/mL, P<0.001), and 28 (6.2 pg/mL, P<0.001) than before transplantation (median value of 2.4 pg/mL). Importantly, IL-7 and IL-15 levels on days 7 or 14 after transplantation did not predict grade II–IV acute GVHD.

**Conclusions:**

These data suggest that IL-7 and IL-15 levels remain relatively low after nonmyeloablative transplantation, and that IL-7 and IL-15 levels early after nonmyeloablative transplantation do not predict for acute GVHD.

## Introduction

Allogeneic hematopoietic stem cell transplantation (allo-HSCT) following a high dose conditioning regimen has been the best treatment option for many young patients with hematological disorders. The antitumor activity of this approach is based not only on high dose chemo-radiotherapy given in the conditioning regimen but also on immune-mediated graft-versus-tumor effects [1], [2]. These observations are the basis of the development of allo-HSCT following nonmyeloablative conditioning, in which eradication of malignant cells depends on graft-versus-tumor effects [3]–[6].

T-cell recovery after allo-HSCT following high-dose conditioning depends on both homeostatic peripheral expansion (HPE) of donor T cells contained in the graft, and T cell neo-production from donor hematopoietic stem cells (thymo-dependent pathway) [7]–[15]. In young patients given myeloablative allo-HSCT, most circulating T cells during the first months following HSCT are the progeny of T cells infused with the grafts [16], while neogeneration of T cells by the thymus plays an increasing role in reconstituting the T cell pool beyond day 100 after allo-HSCT [17]–[22]. Since HPE allow the expansion of both NK cells and non-tolerant T cells, it is generally accepted that HPE is one of the driving force of graft-versus-tumor effects.

Several studies have demonstrated that IL-7 and IL-15 are the main driving forces of HPE after allo-HSCT following high-dose conditioning [7], [23]. IL-7 is a γ-common chain cytokine that is secreted by stromal cells from multiple organs including thymus, bone marrow, and lymphoid organs. IL-7 is required for human T cell development since mutations in the IL-7 receptor alpha can lead to severe combined immunodeficiency [24]. Administration of IL-7 has been shown to dramatically increase peripheral T cell numbers, primarily through augmentation of HPE [25]–[31]. IL-15 is another γ-common chain cytokine secreted by antigen-presenting cells, bone marrow stroma, thymic epithelium, and epithelial cells in the kidney, skin, and intestines [32]. IL-15 plays an important role in the development and function of NK cells, and of NK/T cells, and is required for optimal proliferation of CD8^+^ T cells and for homeostatic proliferation of CD8^+^ memory T cells [33]–[39].

While high-dose conditioning regimens typically induce a profound lymphodepletion, progressive replacement of host-derived T cells by donor-derived T cells is the rule after nonmyeloablative conditioning [40], [41]. This prompted us to analyze the kinetics of IL-7 and IL-15 blood levels after allo-HSCT following a nonmyeloablative conditioning with the aim of determining whether there is a rational for boosting HPE and perhaps graft-versus-tumor effects in patients with high risk disease given grafts after nonmyeloablative conditioning by administering IL-7 and/or IL-15.

## Patients and Methods

### Patients and Donors

Data from 70 patients transplanted between March 2007 and April 2011 at the University of Liège were included in the study (Table 1). All patients were given G-CSF-mobilized peripheral blood stem cells (PBSC) after low-dose [2 Gy (n = 60), or 4 Gy (n = 10)] total body irradiation (TBI)-based nonmyeloablative regimen. Twenty-three nonmyeloablative recipients who were given PBSC from HLA-mismatched unrelated donors were co-transplanted with third party mesenchymal stromal cells (MSCs) as a potential way to prevent severe GVHD [42]. Further, 3 nonmyeloablative recipients were included in a double blind randomized study assessing the impact of MSC co-transplantation on transplantation outcomes. No patient was given in-vivo T cell depletion.

**Table 1:** Patients’ characteristics.

	Nonmyeloablative conditioning (n = 70)
Median age (range)	50 (16–73)
Gender (male/female)	48/22
Diagnostic (# of patients)	
Acute myeloid leukemia in CR	21
Acute lymphoblastic leukemia in CR	4
Chronic myeloid leukemia	1
Chronic lymphocytic leukemia	6
Lymphoma	16
Myelodysplatic syndrome/myeloproliferative disorder	9
Multiple myeloma	13
Donor (# of patients)	
Sibling	13
Unrelated	57
Conditioning regimen (# of patients)	
TBI 2 Gy	1
Fludarabine 90 mg/m^2^+TBI 2 Gy	59
Fludarabine 90 mg/m^2^+TBI 4 Gy	10
Immunosuppressive regimen (# of patients)	
Tacrolimus+MMF	70
Co-transplantation with MSC	
Yes	23
No	44
Unknown*	3
Graft composition; median (range) x 10^6^/kg	
CD34	5.4 (1.1–14.5)
CD3	314 (92–1216)

* double blind randomized study: The information of which of these 3 patients (if any) have been given MSC has been given by the director of the Cell Laboratory only to LS (the statistician); TBI, total body irradiation; MMF, mycophenolate mofetil.

### Ethics

Written informed consent was obtained from each patient to undergo allo-HSCT and to collect, store and analyze blood samples for research purposes. The Ethics Committee of the University of Liège (“Comité d’Ethique Hospitalo-Facultaire Universitaire de Liège”) approved the consent form as well as the current research study protocol (protocol #B707201112193).

### Clinical Management

The clinical management has been performed as previously reported [43], [44]. Chimerism levels among peripheral T-cells were generally measured with PCR-based analysis of polymorphic microsatellite regions (AmpFlSTR® Identifiler®, Applied Biosystems, Lennik, Belgium) [43]. CD3 (T-cell) selection was carried out with the RosetteSep^R^ human T-cell enrichment kit (StemCell Technologies, Vancouver, Canada) [43], [44].

### Cytokines Levels

EDTA-anticoagulated plasma and serum samples were obtained before conditioning and about once time per week after transplantation until day 100. Samples were aliquoted and stored at −80°C within 3 hours after collection until measurement of cytokines. Kinetic courses of IL-7 production in plasma samples were evaluated before conditioning and approximately at days 7, 14, 28, 40, 60, 80 and 100 after allo-HSCT. IL-15 serum sample levels were assessed before conditioning and approximately at days 7, 14 and 28 after allo-HSCT. IL-7 and IL-15 levels were measured by ELISAs following the manufacturer’s protocol (High sensitivity IL-7 and IL-15 quantikine, R&D Systems, Minneapolis, MN, USA). The standard curve ranges for IL7 were 0.25 to 16 pg/mL, and the minimal detectable dose was <0.1 pg/mL. No patient had IL-7 levels below this threshold in the current study. The standard curve ranges for IL15 were 3.9 to 250 pg/mL, and the minimal detectable dose was <2 pg/mL. Il-15 levels were between 0 and 2 pg/mL in our study in 15 patients before transplantation, in no patient on days 7 and 14, and in 1 patient on day 28. No sample dilution was performed for IL-15 assay. For IL-7 analysis, samples were diluted twice. Patient samples whose cytokine level were out of standard curve range, were re-assessed after dilution.

### Immune Recovery

Immune recovery was prospectively assessed as previously described [43], [44]. Briefly, patients’ peripheral white blood cells were phenotyped using 4 color flow cytometry after treatment with a red blood cell lyzing solution. The following antibodies were used: CD3-ECD (Beckman Coulter, Iotest #A07748); CD4-V450 (Becton Dickinson Horizon #560345); CD8-FITC (Beckman Coulter Iotest #A07756); CD56-PC7 (Beckman Coulter Iotest #A21692); CD45RA-PE (Dako #R7086). The percentage of positive cells was calculated relative to total nucleated cells, after subtraction of non-specific staining. Absolute counts were obtained by multiplying the percentages of positive cells by the white blood cell counts (XE-5000 hematology analyzer, Sysmex, Kobe, Japan). Absolute lymphocytes counts (ALC) were measured directly by the XE-5000 analyzer or after microscopic review of the blood smears when the automated differential was flagged. Absolute white blood cell counts were used instead of ALC when white blood cell counts were below 150 cells ×10^9^/L.

### Statistical Analyses

The Mann Whitney test was used to compare counts of lymphocyte subset and cytokine levels in patients given grafts after 2 Gy or 4 Gy TBI. The Wilcoxon matched pair test was used to compare cytokines levels before and at various time points after transplantation. Generalized linear mixed models were used to analyze factors affecting immune recovery and cytokine levels after transplantation. Factors included in the models included : (1) dose of TBI (2 Gy vs 4 Gy), MSC infusion or not, number of days after allo-HSCT, number of CD3^+^ cells transplanted, donor type (related vs unrelated), patient age, and donor age for analyses examining lymphocyte counts; (2) dose of TBI (2 Gy vs 4 Gy), MSC infusion or not, grade II–IV acute GVHD the first 100 days after transplantation, number of CD3^+^ cells transplanted, donor type (related vs unrelated), patient age, and donor age, and either IL-7 or IL-15 levels on days 7–14 (median) for analyses examining lymphocyte count increments from days 14–28 (median) to days 80–100 (median); and (3) number of days after allo-HSCT, number of CD3^+^ cells transplanted, donor type (related vs unrelated), dose of TBI (2 Gy vs 4 Gy), ALC, CRP levels, donor and patient ages, and MSC infusion or not, for analyses of cytokine levels. Incidences of acute GVHD according to the cytokines levels were assessed using cumulative incidence methods. A Cox model was constructed for determining potential factors associated with the occurrence of grade II–IV acute GVHD the first 200 days after transplantation. Factors included in the model included median day 7 and day 14 IL-7 levels, median day 7 and day 14 IL-15 levels, dose of TBI (2 Gy vs 4 Gy), donor type (related vs unrelated), female donor to male recipient versus other gender combination, MSC infusion or not, patient age, and donor age. Spearman’s correlation was used to examine the relationship between parameters. Statistical analyses were carried out with Graphpad Prism (Graphpad Software, San Diego, CA) and SAS version 9.2 for Windows (SAS Institute, Cary, NC, USA).

## Results

### Immune Recovery

**Figure 1:**
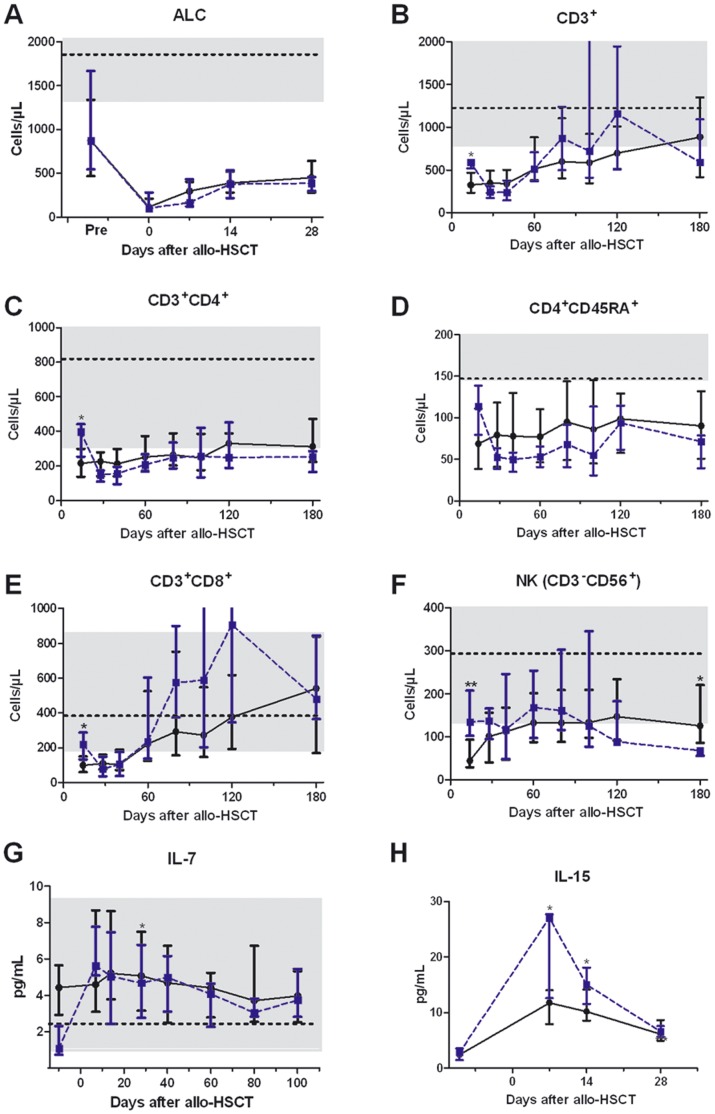
Median ALC (A), median MNC-subset cell counts (B–F), and median IL-7 (G) and IL-15 (H) after allogeneic hematopoietic cell transplantation following 2 Gy (continuous line) or 4 Gy (broken line) total body irradiation. The error bars shows the 25^th^ and 75^th^ percentiles. For ALC and MNC-subset, horizontal lines show the medians and the grey square the limit of normal value (if non truncated) in 47 healthy volunteer donors; for IL-7, horizontal line shows the medians and the grey square the limit of normal value according to the manufacturer brochure. *, P<0.05; **, P<0.01; ***, P<0.001.

Median ALC count on day 0 was 110 (range, 10–5440) cells/µl, demonstrating the persistence of recipient T cells at the time of transplantation. While median CD8^+^ T cell levels reached the lower limit of normal values from day 60 after transplantation, median CD4^+^ T cell (including naïve CD4^+^ T cells) remained below the lower limit of normal values the first 6 months after transplantation (Figure 1). No significant difference of T cell subset counts were observed between 2 Gy and 4 Gy TBI regimen. Using generalized linear mixed models taking into consideration data from day 14, 28, 40, 60, 80 and 100 for each patient, counts of CD3^+^ T cells (P<0.001), CD8^+^ T cells (P<0.001), CD4^+^ T cells (P = 0.024), NK cells (P<0.001) and NK/T cells (P<0.001) increased over time but not those of naïve CD4^+^ T cells (P = 0.13). Further, high numbers of transplanted CD3^+^ T cells were associated with higher counts CD3^+^ T cells (P = 0.009), CD8^+^ T cells (P = 0.003), and CD4^+^ T cells (P = 0.0099), while high donor age was associated with lower counts of CD3^+^ T cells (P = 0.04), CD4^+^ T cells (P = 0.05), and naïve CD4^+^ T cells (P = 0.021). There was no significant association between MSC administration and lymphocyte subset counts after transplantation.

### IL-7 Plasma Levels

Median IL-7 plasma levels remained below 6 pg/L throughout the first 100 days (the upper limit of normal range being 9.2 pg/mL (Quantikine© HS catalog number HS750)), although IL-7 plasma levels were significantly higher on days 7 (5.1 pg/mL, P = 0.002), 14 (5.2 pg/mL, P<0.0001) and 28 (5.1 pg/mL, P = 0.03) (but not thereafter) than before transplantation (median value of 3.8 pg/mL) (Figure 1G). Using generalized linear mixed models, low number of transplanted CD3^+^ T cells (P = 0.001), low ALC level the day of IL-7 assessment (P<0.0001), high donor age (P = 0.003), having received PBSC from unrelated donors (p = 0.006), and high level of CRP the day of IL-7 assessment (*P* = 0.033) were associated with high levels of IL-7 (Table 2).

**Table 2:** Multivariable analyses of factors affecting cytokines levels on days 7 and 14 after allo-HSCT.

	Factor(s) associated with higher levels* ^,^ †
IL-7	- Low ALC on day 7 or 14 (P<0.001).
	- Low # of transplanted T cells (CD3^+^) (P = 0.001).
	- High CRP levels on day 7 or 14 (P = 0.033).
	- Unrelated donors (P = 0.006).
	- High donor age (P = 0.003).
IL-15	- 4 vs 2 Gy TBI (P = 0.002).
	- Unrelated donors (P = 0.001).
	- High CRP levels on day 7 or 14 (P = 0.006).
	- Low ALC on day 7 or 14 (P<0.001).

* Other factors assessed were number of days after allo-HSCT, patient age, and mesenchymal stromal cells infusion or not;

† P values were determined according to generalized linear mixed models;

TBI, total body irradiation.

### Il-15 Serum Levels

Median IL-15 serum levels were significantly higher on days 7 (12.5 pg/mL, P<0.001), 14 (10.5 pg/mL, P<0.001) and 28 (6.2 pg/mL, P<0.001) than before transplantation (median value of 2.4 pg/mL) (Figure 1H). IL-15 levels on day 7 and 14 were significantly higher in 4 Gy than 2 Gy TBI. Using generalized linear mixed models, conditioning with 4 versus 2 Gy TBI (P = 0.002), having received PBSC from unrelated donors (P = 0.001), low ALC level the day of IL-15 assessment (P<0.001), and high level of CRP the day of IL-15 assessment (P = 0.006) were each associated with high IL-15 levels on days 7 and 14 after allo-HSCT (Table 2).

### Correlation between IL-7 and IL-15 Levels and Lymphocyte Subset Counts on Days 14 or 28 after allo-HSCT

**Figure 2:**
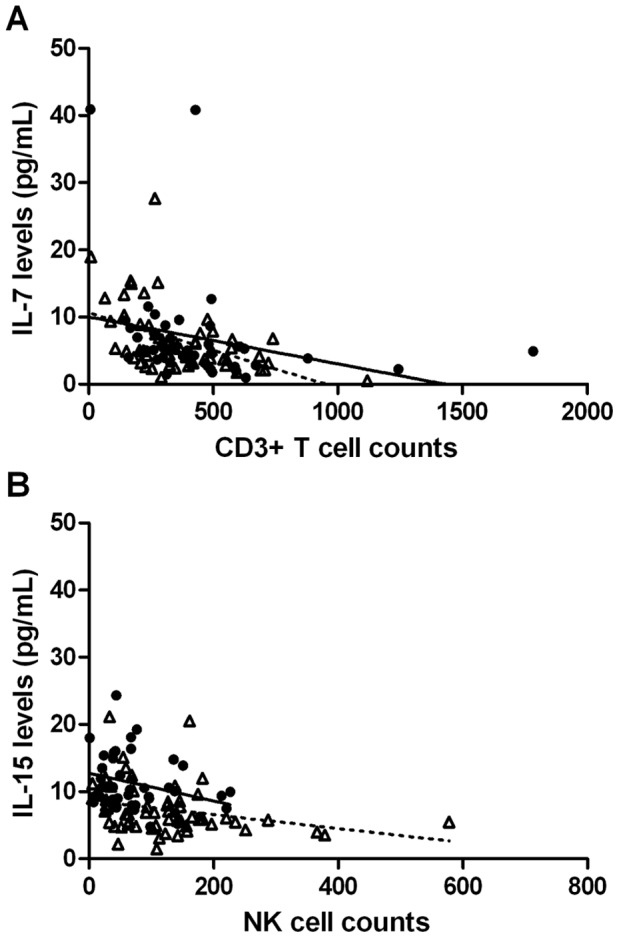
Correlation between CD3+ T cell counts and IL-7 levels on day 14 (black circles and continuous line) and on day 28 (open triangles and broken lines) after transplantation (A). Correlation between NK cell counts and IL-15 levels on day 14 (black circles and continuous line) and on day 28 (open triangles and broken lines) after transplantation (B).

Day 14 IL-7 levels inversely correlated with day 14 counts of CD3^+^ T cells (R = −0.46, P = 0.002; Figure 2A), CD8^+^ T cells (R = −0.41, P = 0.006), CD4^+^ T cells (R = −0.44, P = 0.004), and memory CD4^+^ T cells (R = −0.45, P = 0.003), but not with counts of naïve CD4^+^ T cells (R = −0.28, P = 0.07), NK/T cells (R = −0.04, P = 0.8) nor NK cells (R = −0.14, P = 0.4). There was a weak association between day 14 IL-7 and IL-15 levels (R = 0.27, P = 0.049). Further, day 14 IL-15 levels correlated with day 14 counts of NK cells (R = −0.32, P = 0.039; Figure 2B) and of NK/T cells (R = −0.32, P = 0.037), but not with those of other T cell subsets.

Day 28 IL-7 levels inversely correlated with day 28 counts of CD3^+^ T cells (R = −0.47, P<0.001; Figure 2A), CD8^+^ T cells (R = −0.41, P = 0.002), CD4^+^ T cells (R = −0.39, P = 0.002), naïve CD4^+^ T cells (R = −0.40, P = 0.002), and memory CD4^+^ T cells (R = −0.38, P = 0.004), but not with counts of NK/T cells (R = −0.17, P = 0.2), nor NK cells (R = −0.02, P = 0.9), nor with day-28 donor T cell chimerism levels (R = 0.0, P = 0.95). There was no significant association either between day 28 IL-7 and IL-15 levels (R = 0.07, P = 0.6). Further, day 28 IL-15 levels correlated with day 28 counts of NK cells (R = −0.32, P = 0.015; Figure 2B) but not with those of T cell subsets, nor with day-28 donor T cell chimerism levels (R = 0.14, P = 0.29).

To further assess the potential association between early IL-7 or IL-15 levels on immune recovery, we analysed whether there was a relationship between median cytokine levels on days 7 and 14 and the difference of lymphocyte subset counts between days 80–100 (median) and days 14–28 (median). Interestingly, in multivariate analyses, early IL-7 levels did not correlate with any lymphocyte subset increment from days 14–28 to day 80–100 after transplantation, while high IL-15 levels early after transplantation correlated with a lower increment of NK cells over time (P = 0.04).

### IL-7 and IL-15 Levels did not Predict for Subsequent Acute GVHD

**Figure 3:**
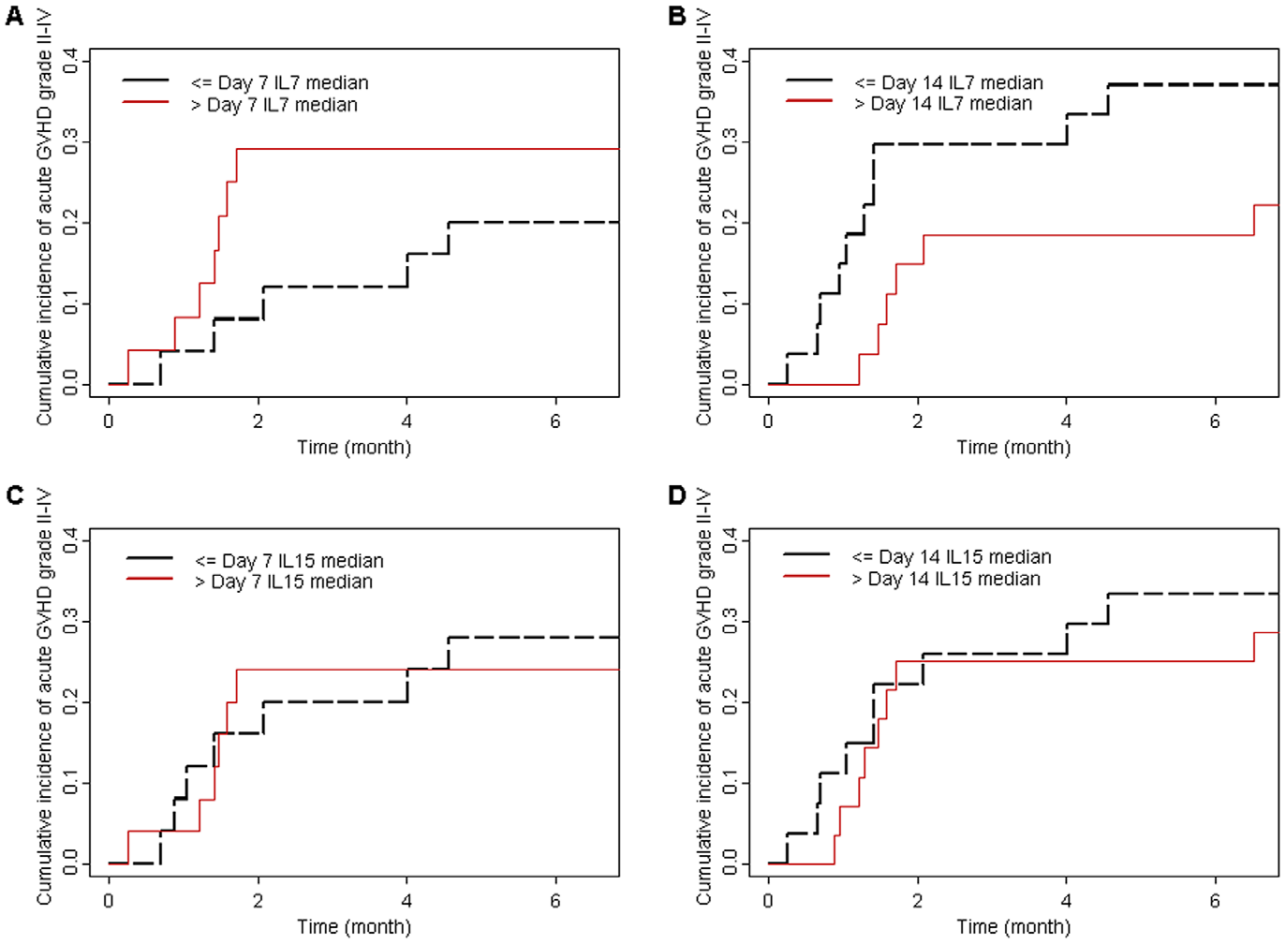
Cumulative incidence of grade II–IV acute GVHD according to day 7 IL-7 plasma levels among nonmyeloablative recipients (P = 0.4) (A). Cumulative incidence of grade II–IV acute GVHD according to day 14 IL-7 plasma levels among nonmyeloablative recipients (P = 0.18) (B). Cumulative incidence of grade II–IV acute GVHD according to day 7 IL-15 serum levels among nonmyeloablative recipients (P = 0.8) (C). Cumulative incidence of grade II–IV acute GVHD according to day 14 IL-15 serum levels among nonmyeloablative recipients (P = 0.6) (D).

The 180-day cumulative incidence of grade II–IV acute GVHD was 30%, a rate similar to what has been observed by other group of investigators using similar conditioning regimen [45]. As shown in the Figure 3, no statistically significant association between cytokines levels on days 7 or 14 after transplantation and occurrence of grade II–IV acute GVHD were observed.

Specifically, the 180-day cumulative incidence of grade II–IV acute GVHD was 29% in patients with day 7 IL-7 levels>median (5.1 pg/mL) versus 20% in patients with day 7 IL-7 levels ≤ median (P = 0.38) (Figure 3A). Similarly, the 180-day cumulative incidence of grade II–IV acute GVHD was 19% in patients with day 14 IL-7 levels>median (5.2 pg/mL) versus 37% in patients with day 14 IL-7 levels ≤ median (P = 0.18) (Figure 3B).

The 180-day cumulative incidence of grade II–IV acute GVHD was 24% in patients with day 7 IL-15 levels>median (12.5 pg/mL) versus 28% in patients with day 7 IL-15 levels ≤ median (P = 0.8) (Figure 3C). Similarly, the 180-day cumulative incidence of grade II–IV acute GVHD was 25% in patients with day 14 IL-15 levels>median (10.5 pg/mL) versus 33% in patients with day 14 IL-15 levels ≤ median (P = 0.8) (Figure 3D).

Finally, in a multivariate Cox model, neither median IL-7 levels (P = 0.17 with a trend for an inverse correlation) on days 7–14 nor median IL-15 levels (P = 0.21 with a trend for a positive correlation) on days 7–14 correlated with occurrence of grade II–IV acute GVHD the first 200 days after transplantation. Similarly, the use of MSC was not associated with decreased incidence of grade II–IV acute GVHD. This could be explained by the fact that all 23 MSC recipients versus of 9 of the remaining 49 patients (18%) received PBSC from HLA-mismatched donors. None of the other factors tested (dose of TBI, donor type, female donor to male recipient versus other gender combination, patient age, and donor age) were significantly associated with the incidence of grade II–IV acute GVHD in the current study.

### IL-15 Levels did not Predict for Subsequent Relapse/Progression

Given that a previous publication showed an association between high IL-15 levels and low risk of relapse/progression [46], we compared the cumulative incidence of relapse/progression according to IL-15 levels 14 days after transplantation in our cohort of patients. The 6-month and 1-year cumulative incidences of relapse/progression were 29% and 32%, respectively, in patients with day 14 IL-15 levels>median (10.5 pg/mL) versus 37% and 46%, respectively, in patients with day 14 IL-15 levels ≤ median (P = 0.57).

## Discussion

Following allo-HSCT, eradication of residual tumor cells depends in part (in case of high-dose conditioning) or mainly (in case of nonmyeloablative conditioning) on immune-mediated graft-versus-tumor effects [1], [2], [4]. Prior studies have demonstrated a close relationship between T cell reconstitution and graft-versus-tumor effects after allo-HSCT [4], [14], [47]–[49]. Given that HPE allows the expansion of potentially alloreactive T cell clones, it has been generally accepted that HPE plays a major role in graft-versus-tumor effects, but could also cause or favor acute GVHD. This prompted us to investigate the kinetics of IL-7 and IL-15 levels in a cohort of 70 patients given grafts after truly nonmyeloablative conditioning.

First, patients given grafts after nonmyeloablative conditioning had only a modest (<2 fold) increase of IL-7 levels after transplantation (contrarily to what we observed in another cohort of patients given grafts after myeloablative conditioning [50]), that persisted up to day 21. This is probably due to the fact that nonmyeloablative patients experienced relatively mild lymphopenia (and thus continue to consume the IL-7 produced by stromal cells) as demonstrated by the persistence of median ALC counts of 110 cells/µL at the time of transplantation. Although the first T cell chimerism assessment in current patient was usually around day 28 after HSCT, a prior study analyzing data from patients given similar conditioning regimen demonstrated that a median of 50 CD3^+^ T cells of recipient origin/µL persisted on day 14 after HSCT [40]. Further, as observed by other groups of investigators [46], [51], [52], there was a strong inverse correlation between IL-7 levels and absolute lymphocyte counts [46], [52], as well as a strong inverse correlation between IL-7 levels and T cell subsets on days 14 and 28 after transplantation. Other factors associated with IL-7 levels included high CRP levels, and low numbers of transplanted T cells. Levels of IL-7 in current nonmyeloablative recipients where lower to what was observed by Thiant *et al.* in a cohort of 45 patients given grafts after fludarabine +2 Gy TBI (n = 18) or more intense but still reduced-intensity conditioning (n = 27) [52], and where much lower than what was observed by Dean *et al.* in patients given grafts after sequential chemotherapy followed by a chemotherapy/fludarabine-based reduced-intensity conditioning [53]. This apparent discrepancy is probably explained the fact than median ALC counts on day 0 were 110 (range, 10–5440) cells/µl in current patient versus 0 (range, 0–322) cells/µL in the Dean *et al.* study, while median counts of CD3^+^ T cells were 0 (range, 0–1900) cells/µL at the time of transplantation in Thiant *et al.* study.

Il-15 levels were lower in nonmyeloablative patients conditioned with 2 Gy TBI than in those conditioned with 4 Gy TBI, demonstrating that the release of IL-15 was proportional to the intensity of the conditioning regimen. As observed by Thiant *et al.* [46], [52], there was a correlation between IL-7 and IL-15 levels on day 14 (but not on day 28) after transplantation, and an inverse correlation between IL-15 levels and NK cell counts. Other factors affecting IL-15 levels included high CRP levels.

Several observations demonstrate that immune recovery depended mainly on HPE the first year after nonmyeloablative conditioning regimen in current patients. Firstly, there was a strong correlation between the number of infused T cells and high counts of CD4^+^ and CD8^+^ T cells, as previously observed [43], [54]. Secondly, thymic function was minimal during the first 100 days after allo-HSCT given that levels of naïve CD4^+^ T cells did not significantly increase the first 100 days after transplantation despite that some naïve T cells can undergo HPE and keep their naïve phenotype. Third, there was a correlation between high donor age and low counts of CD3^+^ T cells (P = 0.04), CD4^+^ T cells (P = 0.05), and naïve CD4^+^ T cells (P = 0.021), as previously observed in patients given grafts after nonmyeloablative conditioning [55]. Despite that, we failed to find any significant association between IL-7 and/or IL-15 levels early after transplantation and increment of T cell subset counts from days 14–28 to day 80–100, even after adjusting for potentially confounding cofactors.

A number of previous studies have demonstrated that high levels of IL-7 [46], [52], [53] and/or IL-15 [46], [52] early after transplantation correlated with subsequent occurrence of grade II–IV acute GVHD, while others study failed to find such an association [51], [56]. The largest study including data from 153 consecutive allogeneic transplant recipients given grafts after high-dose conditioning and ATG observed no correlation between IL-7 levels early after transplantation and acute GVHD, while, interestingly, there was an inverse correlation between IL-15 levels early after transplantation and grade II–IV acute GVHD [57]. Further, a recent study demonstrated that administration of IL-7 after allogeneic T cell-depleted transplantation in humans did not increase acute GVHD [58]. In the current study, we did not observe any association between levels of IL-7 or IL-15 early after allo-HSCT and grade II–IV acute GVHD. The same was true after adjusting the analyses for potentially confounding cofactors. Differences in postgrafting immunosuppression might be the cause for these apparent discrepancies between studies. As example, it has been shown that tacrolimus (given in patients included in the current study) decreased T cell proliferation induced by IL-7 [59], and tacrolimus levels were kept high in our patients the first weeks after transplantation (median 18.6, 16.4, 14.9 and 14.3 µg/L on days 0, 7, 14 and 21 after transplantation, respectively) probably explaining the low relatively incidence of acute GVHD observed [60].

In summary, these data suggest that IL-7 and IL-15 levels remain relatively low after nonmyeloablative transplantation, and that IL-7 and IL-15 levels early after nonmyeloablative transplantation do not predict for acute GVHD.
